# The moderate predictive value of serial serum CRP and PCT levels for the prognosis of hospitalized community-acquired pneumonia

**DOI:** 10.1186/s12931-018-0877-x

**Published:** 2018-10-01

**Authors:** Shuren Guo, Xiaohuan Mao, Ming Liang

**Affiliations:** 1 Department of Clinical Laboratory, The First Affiliated Hospital of Zhengzhou University, East Jianshe Road #1, Zhengzhou, Henan 450002 People’s Republic of China; 2 Key Clinical Laboratory of Henan province, Zhengzhou, Henan People’s Republic of China; 3 Department of Clinical Laboratory, Henan Provincial People’s Hospital, Henan Province Zhengzhou, 450003 People’s Republic of China

**Keywords:** Serial serum CRP, PCT, Predictive value, CAP prognosis

## Abstract

**Background:**

To predict the prognosis by observing the dynamic change of C-reactive protein (CRP) and procalcitonin (PCT) for hospitalized community-acquired pneumonia (CAP).

**Methods:**

The data were collected from January to December 2017 from the first affiliated Hospital of Zhengzhou University. Demographic and clinical patient information including age, length of hospital stay and Charlson Comorbidity Index (CCI) were recorded. Blood samples were taken for CRP, PCT, and white blood cell count (WBC). Receiver Operating Characteristic (ROC) curve was used to verify each biomarker’s association with the prognosis of pneumonia.

**Results:**

A total of 350 patients were enrolled in the study. The 30-day mortality was 10.86%. Serial serum CRP3, CRP5, PCT3, PCT5 and PCT5c levels were statistically lower in CAP survivors than non-survivors. CRP3c < 0, CRP5c < 0 and PCT5c < 0 were observed with a statistically lower frequency in patients with 30-day mortality and initial treatment failure. The AUC for 30-day mortality for serial CRP levels combined with CRP clearances was 0.85 (95% CI 0.77–0.92), as compared to an AUC of 0.81 (95% CI 0.73–0.9) for serial PCT levels combined with PCT clearances.

**Conclusions:**

Serum serial CRP and PCT levels had moderate predictive value for hospitalized CAP prognosis. The dynamic CRP and PCT changes may potentially be used in the future to predict hospitalized CAP prognosis.

**Electronic supplementary material:**

The online version of this article (10.1186/s12931-018-0877-x) contains supplementary material, which is available to authorized users.

## Background

Diagnosis of pneumonia in critically ill patients is usually challenging. Signs and symptoms with enormous heterogeneity, such as dyspnea, may be non-diagnostic or atypical, chest X-ray results may be uncertain, also complications may be confounding factors [1–3]. Thus, biomarkers of inflammation or infection, such as procalcitonin (PCT) and C-reactive protein (CRP), have been proposed as a guide in the diagnostic process [4–6]. Elevated serum PCT and CRP were associated with community-acquired pneumonia and ventilator-associated pneumonia (VAP) [5, 7].

CRP is a well-established biomarker in many clinical settings, but has been traditionally considered insufficient as a useful marker in the diagnosis of pneumonia. In fact, all infections, stress reactions, autoimmunity and tumor disease can contribute to the increase in serum CR*P* values [8].

PCT is a 116-amino acid long precursor of calcitonin, which is produced by the thyroid. In sepsis, macrophages and the monocytic cells of the liver are involved in the synthesis of PCT,which is elevated in sepsis [9, 10]. The degree of induction of PCT correlates with the severity of systemic infection and the presence of organ dysfunction.

Due to multiple confounding factors, several studies have reported controversial results on the role of CRP and PCT in the diagnosis of pneumonia in multiple elderly patients [1, 11, 12]. The importance of serum CRP and PCT levels on diagnosis is well established [5, 7, 13, 14], The mean values of certain cytokines are statistically different from patients with treatment failure vs patients without treatment failure, the wide range of values for particular cytokines make it difficult to use the value of a single patient to predict clinical outcomes. A dynamic approach of assessing biomarkers may provide additional survival information. Markers of the inflammatory response and their kinetics have been studied in the prediction of outcomes in sepsis [15] and VAP [16, 17]. As reported by Huang MY, et al., PCT clearance (PCTc) has been introduced in a previous studies as a tool for monitoring the changes of PCT levels during severe sepsis [18, 19]. Similar to PCTc in the previous study, in our study we introduced CRP clearance (CRPc) to monitor the changes of CRP levels during the treatment of hospitalized CAP. Since PCTc and CRPc measures the relative changes in PCT and CRP to the baseline levels, they are postulated to be a better predictor of prognosis. However, both PCTc and CRPc are not common in clinical practice.

Therefore, the hypothesis of this study is whether CRP and PCT levels and their clearance could serve as prognostic biomarkers for hospitalized CAP patients. The aim of the present study was to evaluate the usefulness of CRP and PCT levels and their clearance as prognostic biomarkers for hospitalized CAP patients.

## Methods

### Study design and patient population

This was a single-center, prospective observational study. Hospitalized pneumonia patients with a radiological confirmation were recruited. The informed consents were obtained from all subjects or their guardians. The study was approved by ethic committee of Zhengzhou University and met the declaration of Helsinki. Diagnosis of CAP required the presence of at least one respiratory symptom in addition to one auscultatory finding or signs of infection (WBC > 10 × 10^9^/L or < 4 × 10^9^/L cells, shivers, core body temperature > 38.0 °C) and a new infiltrate on chest radiograph. The respiratory symptoms included cough, expectoration, dyspnea, tachypnea, or pleuritic chest pain [20]. Radiological findings were verified with results of the real-time PCR tests on blood samples and nasopharyngeal swabs. The Clinical severity of the hospitalized CAP was evaluated by the CURB-65 score, which including confusion, urea, respiratory, and blood pressure plus age > 65 years.

### Measurement of biomarkers

Followed our study design, WBC counts were measured as a part of routine tests using Beckman-coulter LH750 hematology analyzer. Serum CRP and PCT levels were measured on hospital days 1, 3, and 5 in patients. The blood was drawn in vacuum tube filled with separation gel and centrifuged at 3500 rpm for 5 min, and then CRP and PCT were analyzed by Roche cobas 8000 automatic biochemistry analyzer within 30 min. Concentrations of CRP were determined by an immunoturbidimetric assay. The diagnostic cut-off value of CRP was set by manufacturer at 5 mg/L. PCT (ng/mL) levels were measured by electro chemiluminescence immunoassay with a lower limit of detection of 0.02 ng/ml. CRP and PCT levels measured on day 1, day 3 and day 5 were defined as CRP1 and PCT1, CRP3 and PCT3, CRP5 and PCT5, respectively. PCTc was calculated based on the previously reported formula [19], (PCTday3/day5-PCTday1)/PCTday1 × 100% = PCT3c /day5c (%). The calculation of CRPc was referred to the PCTc formula. CRPc on day3 and day5 were abbreviated as CRP3c and CRP5c.

### The detection of CAP pathogen

Viral RNA or DNA was extracted from the respiratory secretions within 24 h, and was then tested using respiratory virus panel (Shanghai ZJ Bio-Tech Co., Ltd) fast assay to detect influenza A/B virus (lot: RR-0226-02), respiratory syncytial virus (RSV)-A (RR-0160-01) and -B (lot:RR-0160-02), parainfluenzavirus-1, − 2, − 3 and − 4 (lot: RR-0156-01,02,03,04), adenovirus (lot:RD-0195-02), human metapneumovirus (hMPV) (lot: RR-0162-02) in accordance with the manufacturer’s instructions.

The autolysin-A (LytA) and wzg (cpsA) genes of *S. pneumoniae* were tested using real-time PCR from blood and swab samples for pneumococcal cases according to the manufacture instructions. *M. pneumoniae* was looked for in blood and nasopharyngeal swabs with nested PCR, as described previously [21]. Routine microbiological examinations were also performed at the Microbiology laboratory and included blood culture, sputum culture, and antigenuria.

### Statistical analysis and data management

Data were analyzed using SPSS v.17.0 software (SPSS Inc., Chicago, IL, USA) for Windows. Frequency comparison was done using the χ2-test. The two-group comparison for continuous data was done with the Mann-Whitney U-test. We used univariate and multivariate logistic regression analysis to study the association between biomarker levels and outcome adjusting the models for the CAP severity score CURB-65 and age. ROC curves were used to evaluate the sensitivity and specificity of PCT and CRP vs pneumonia prognosis. The areas under the curve (AUC) were reported with its 95% confidence interval (CI). All *p*-values were two-tailed and were considered significant for *p* < 0.05.

### Outcomes

The primary endpoint was 30-day mortality and the secondary endpoint was initial treatment failure. Both endpoints were assessed by seven medical students, blinded to the goal and design of the study, by conducting standardized follow-up interviews by telephone at 30 days after baseline. Initial treatment failure was defined as occurring in patients whose antimicrobial agents were changed by the attending physicians because they were ineffective referring to the CAP guideline in China [22]. Serial changes in PCT, CRP, and WBC were analyzed for their potential to estimate the clinical prognosis/outcome.

## Results

### Demographics and clinical presentations

Baseline characteristics of survivors and non-survivors were presented in Table 1. This study included a total of 350 patients with a median age of 58.53 years (58.3% males). The 30-day mortality was found in 10.86% (38/350) of all patients. Patients had a high burden of comorbidities including chronic heart disease (*n* = 100), chronic liver disease (*n* = 22), chronic renal disease (*n* = 47), malignant disease (*n* = 26), Chronic Obstructive Pulmonary Disease (COPD, *n* = 22) and diabetes (*n* = 30). Cough (*n* = 268, 76.6%) and dyspnea (*n* = 249, 71.1%) were the most frequent symptoms. No significant differences of comorbidities and symptoms were found between survivors and non-survivors. CAP was ascribed to bacteria in 176 (50.29%) patients and to one or more viruses in 115 (32.86%) patients (Additional file 1: Table S1). Serial serum CRP3, CRP5, PCT3, PCT5 and PCT5c levels were statistically lower in CAP survivors than non-survivors (Table 1). CRP3c < 0, CRP5c < 0 and PCT5c < 0 were observed with a statistically lower frequency in patients with 30-day mortality (Table 1).

**Table 1:** Characteristics of survivors and non-survivors

	All patients (%) *n*=350	Survivors (%) *n*=312	Non-Survivors (%) *n*=38	*P* value
Age(years)	58.53±19.1	58.59±19.2	58.03±18.9	0.86
Males	204 (58.3)	181(58.0)	23(60.5)	0.7
Comorbidity				
Diabetes Mellitus	30(8.57)	27(8.6)	3(7.8)	0.87
Chronic heart disease	100(28.57)	91(29.1)	9(23.6)	0.48
Chronic liver disease	22(6.29)	20(6.4)	2(5.2)	0.78
Chronic renal disease	47(13.43)	40(12.8)	7(18.4)	0.34
Malignant disease	26(7.43)	24(7.6)	2(5.2)	0.59
History of Shock	17(4.86)	15(4.8)	2(5.2)	0.9
COPD	22(6.29)	18(5.7)	4(10.5)	0.25
Cerebrovascular disease	39(11.14)	35(11.2)	4(10.5)	0.9
Antimicrobial treatment before admission	79(22.6)	70(22.4)	9(23.7)	1
Signs and symptoms				
Cough	268(76.6)	260(83.3)	28(73.6)	0.14
Chest pain	116(33.1)	106(33.9)	10(26.3)	0.34
Expectoration	168(48)	148(47.4)	20(52.6)	0.54
Dyspnea	249(71.1)	221(70.8)	28(73.6)	0.72
Chills	124(35.4)	109(34.9)	15(39.4)	0.58
Headaches	75(21.4)	58(18.5)	17(44.7)	***<0.001***
Myalgia	79(22.6)	71(22.7)	8(21)	0.8
Crackles	114(32.6)	102(32.6)	12(31.5)	0.89
Fever	110(31.4)	96(30.7)	14(36.8)	0.45
Confusion	5(1.4)	1(0.3)	4(10.5)	***<0.001***
CCI class				
0-2	129(36.8)	116(37.1)	13(34.2)	0.7
3-5	180(51.4)	161(51.6)	19(50)	
>5	41(11.7)	35(11.2)	6(15.7)	
CRP1 (mg/L)	65.3±84.7	66.3±85.2	57.1±81.3	0.53
CRP3 (mg/L)	56.4±77.4	50±66.4	109.1±128.4	***<0.001***
CRP3c	556.6±5056.3	575.2±5334.2	401.2±1242.4	0.843
CRP3c<0	223(63.7)	214(68.5)	9(23.6)	***<0.001***
CRP5(mg/L)	44.8±68.5	37.9±61	102.1±96.9	***<0.001***
CRP5c	429.2±3489.6	429.2±3670.1	429.3±1207.6	0.999
CRP5c<0	222(63.4)	213(68.2)	9(23.6)	***<0.001***
PCT1 (ng/mL)	1.8±7.1	1.8±7.3	1.9±5.5	0.96
PCT3 (ng/mL)	1.7±6.3	1.4±4.4	4.1±14.5	***0.012***
PCT3c	791.2±2653.8	793.3±2672.6	774.1±2528.7	0.966
PCT3c<0	174(49.7)	157(50.3)	17(44.7)	0.52
PCT5 (ng/mL)	1.2±3.7	0.8±2	4.3±9.3	***<0.001***
PCT5c	695.3±2463	589.9±2298.2	1555±3454.4	***0.022***
PCT5c<0	179(51.1)	170(54.4)	9(23.6)	***<0.001***
WBC1	10.4±8	10.1±7.2	12.6±12.8	0.081
WBC3	9.5±5	9.2±5	10.8±4.7	0.112
WBC5	10.5±6.6	10.4±7	10.6±4.7	0.932
CURB class				
0-2	289	256	33	0.46
3-5	61	56	5	

Data are presented as means $\overline{x}$ ±SD, or n (%), CRP, C-reactive protein; CURB-65, confusion, urea > 7 mmol/L, respiratory rate≥30 breaths/min, low blood pressure (systolic<90mm Hg or diastolic≤60 mm Hg) and age≥65 years

*PCT* procalcitonin, *COPD* Chronic Obstructive Pulmonary Disease, *SD* standard deviation, *WBC* white blood cell, *CRP3c* 5c: CRP clearance on day3, *day5* PCT3c, *5c* PCT clearance on day 3, day 5

### Statistic analysis for clinical factors and CAP

WBCs, CRP, and PCT levels on hospital days 1, 3, and 5 and their clearance were compared in all groups. The average mean value of these biomarkers comparison is reported in Tables 2, 3 and Additional file 2: Table S2. ANOVA analysis showed that the CAP patients with bacteria pathogens had significantly higher values of CRP and PCT (*P* < 0.05) than those with other causative pathogens (Additional file 2: Table S2).

**Table 2:** Univariate and multivariate analysis of biomarkers for 30-day mortality

	Univariate analysis			Multivariate analysis		
	Odds ratio (95% CI)	Estimate	Univariate *P*-value	Odds ratio (95% CI)	Estimate	Multivariate *P*-value
CRP1 (mg/L)	0.998(0.994–1.003)	−0.001	0.53			
CRP3 (mg/L)	1.006(1.003–1.01)	0.006	< 0.001	1.013 (1–1.025)	0.012	0.002
CRP3c	0.999(0.999–1)	0	0.845			
CRP3c < 0	1.02(0.991–1.049)	0.019	0.174			
CRP5(mg/L)	1.008(1.004–1.01)	0.008	< 0.001	1.011 (1–1.021)	0.011	0.028
CRP5c	1(0.999–1)	0	0.999			
PCT1 (ng/mL)	1.001(0.955–1.05)	0.001	0.96			
PCT3 (ng/mL)	1.036(0.998–1.07)	0.035	0.06			
PCT3c	0.999(0.999–1)	0	0.966			
PCT5 (ng/mL)	1.21(1.08–1.357)	0.191	< 0.001	1.277 (1.004–1.624)	0.244	0.046
PCT5c	1(0.999–1)	0	0.052			
WBC1	1.025(0.993–1.059)	0.025	0.117			
WBC3	1.061(0.985–1.143)	0.059	0.118			
WBC5	1.004(0.906–1.113)	0.004	0.931

**Table 3:** Univariate and multivariate analyses of biomarkers for initial treatment failure

	Initial treatment failure		Univariate Odds ratio(95% CI)	Univariate *P*-value	Multivariate Odds ratio(95% CI)	Multivariate *P*-value
	Yes	No				
CRP1 (mg/L)	**71±88.7**	**64±83.9**	**0.999(0.996-1.003)**	**0.55**	**1.008(1.001-1.013)**	**0.009**
CRP3 (mg/L)	**89.2±107.6**	**48.9±66.8**	**0.995(0.991-0.998)**	**0.001**	**0.992(0.985-0.999)**	***0.035***
CRP3c	801.8±3644.9	501±5328.5	1	0.673	1(0.999-1)	0.33
CRP5(mg/L)	79.7±96.3	37.2±58.3	0.993(0.989-0.997)	<0.001	0.996(0.989-1.001)	0.15
CRP5c	682.9±2888.4	371.7±3614.2	1	0.534	1(0.999-1)	0.299
PCT1 (ng/mL)	4.6±13.6	1.2±4.2	0.936(0.892-0.983)	0.009	0.89(0.82-0.965)	***0.005***
PCT3 (ng/mL)	**2.8±11.2**	**1.4±4.5**	**0.975(0.941-1.01)**	**0.163**	**1.134(1.017-1.263)**	***0.022***
PCT3c	469.1±1972.7	865.2±2784.4	1	0.293	1(0.999-1)	0.403
PCT5 (ng/mL)	**2.8±7.3**	**0.8±2.1**	**0.868(0.892-0.983)**	**0.005**	**0.851(0.751-0.963)**	***0.01***
PCT5c	1013.8±2761.2	622.1±2388.7	1	0.268	1(0.999-1)	0.658

a Variable(s) entered on step 1: CRP1, CRP3, CRP3c, CRP5, CRP5c, PCT1, PCT3, PCT3c, PCT5, PCT5c, and CURB65

We used univariate and multivariate logistic regression models to investigate associations between serum biomarker levels and outcome (Table 2). In univariate analysis, no significant association of CRP1 [OR (95% CI): 0.998(0.994–1.003)] and PCT1 levels [OR (95% CI): 1.001(0.955–1.05)] or WBC counts with 30-day mortality was found. Significant predictive ability was found for 30-day mortality with CRP3 [OR (95% CI): 1.006(1.003–1.01)], CRP5 [OR (95% CI): 1.008(1.004–1.01)] and PCT5 [OR (95% CI): 1.21(1.08–1.357)] levels respectively. The significance did not disappear after adjust for age, sex and CURB-65 in multivariate logistic regression model.

This study did not show that patients with initial treatment failure had significant higher CRP1 levels than others (71 vs 64, *P* = 0.55). On the other hand, patients with initial treatment failure had significantly higher levels of CRP3, CRP5, PCT1, PCT3 and PCT5 than others (Table 3), which indicated that serial measurements of these serum biomarker levels were also useful for predicting whether initial CAP treatment would be successful.

### Correlation between PCT and CRP and their clearance

Assessment of correlation between biomarkers was performed by Spearman’s rank correlation analysis. Table 4 showed correlations of CRP, PCT and their clearance in the overall population. At baseline, day 3 and day 5, we found significant correlations between PCT and CRP, no correlations were found for PCT3c and CRP3c (R^2^ = 0.09, *P* = 0.11). However, the maximum correlation coefficient was 0.35, which is smaller than 0.8, indicating the low level of multicollinearity among each biomarker.

**Table 4:** Correlation of biomarkers characteristics at different time

	PCT1(ng/ml)	PCT3(ng/ml)	PCT5(ng/ml)	PCT3c	PCT5c
CRP1 (mg/L)	*R*^*2*^=0.35 *P*=0.0001^*^				
CRP3 (mg/L)		*R*^*2*^=0.19 *P*=0.0001^*^			
CRP3c				*R*^*2*^=0.09 *P*=0.11	
CRP5(mg/L)			*R*^*2*^=0.21, *P*=0.0001^*^		
CRP5c					*R*^*2*^=0.17 *P*=0.002^*^

Correlation is significant at the 0.01 level (2-tailed)

^*^Pearson Correlation was used to test the correlation between biomarkers

### Prognostic accuracy of serial values of PCT and CRP

**Fig. 1:**
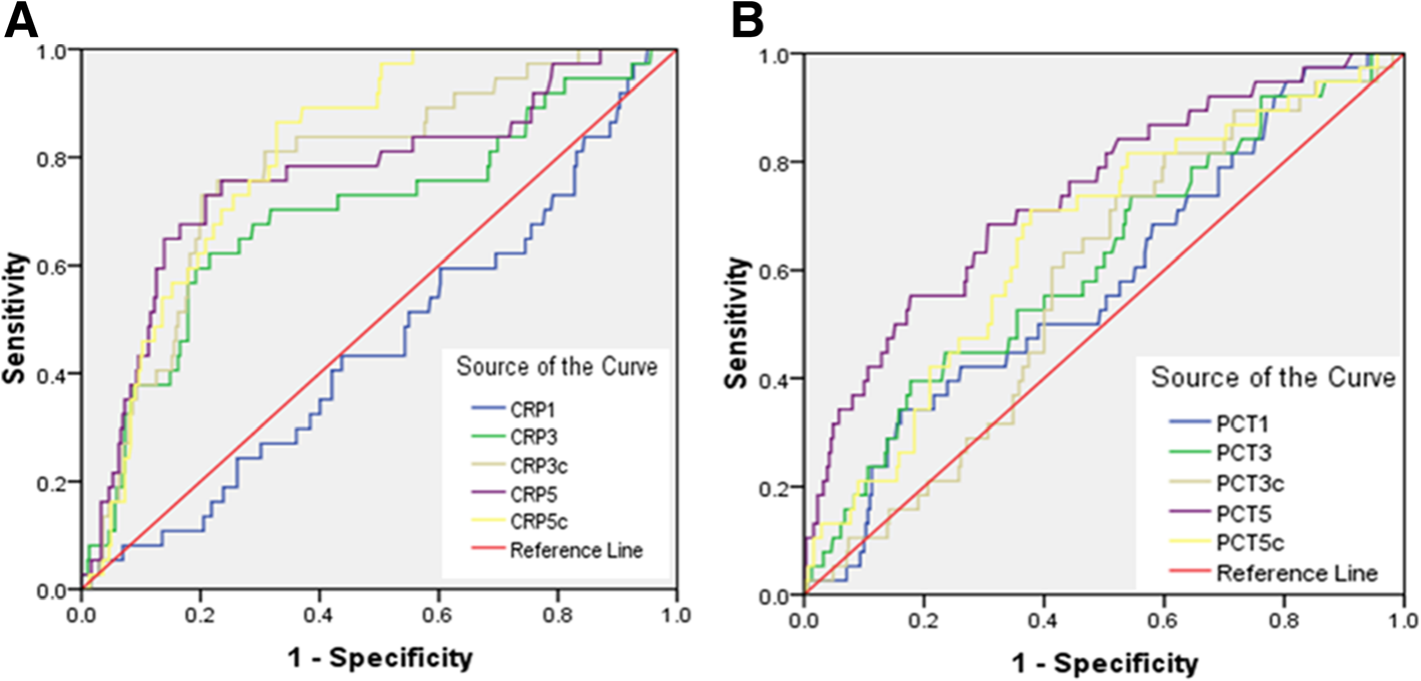
ROC curve of CRP, PCT levels and their clearance vs pneumonia prognosis

**Fig. 2:**
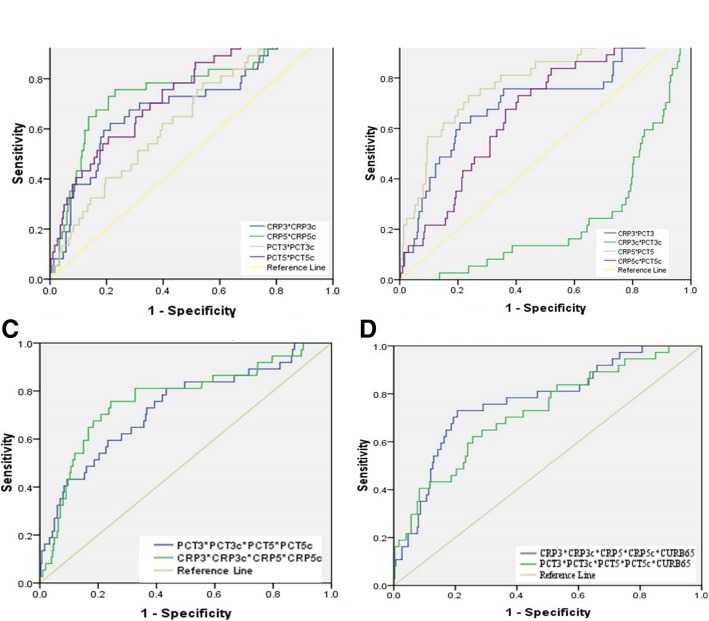
Prognostic performances of Biomarkers and CURB-65 in predicting pneumonia prognosis

Table 5 showed the ROC curve of each biomarker and each biomarker combined. For the single biomarker, the peak areas under the ROC curve of CRP5c and PCT5 to predict 30-day mortality was 0.81 (95%CI: 0.75–0.87; *P <* 0.001) and 0.73 (95%CI: 0.65–0.82; *P <* 0.001), respectively (Table 5, Fig. 1a, b). The capacity of serial serum biomarkers combined to predict 30-day mortality was higher than only one biomarker or a combination of two of the biomarkers. The AUC for 30-day mortality for serial CRP levels combined with CRP clearances was 0.85 (95% CI 0.77–0.92), as compared to an AUC of 0.81 (95% CI 0.73–0.9) for serial PCT levels combined with PCT clearances. Furthermore, their AUC-ROC did not increase if they were used in combination with CURB65 (Table 5, Fig. 2c, d).

**Table 5:** Prognostic performance of Biomarkers and CURB-65 in predicting pneumonia prognosis

Variable(s)	AUC	SE	*P* value	95%CI
CRP1 (mg/L)	0.45	0.05	0.37	0.36-0.55
CRP3 (mg/L)	0.69	0.05	<0.001	0.6-0.8
CRP3c	0.77	0.04	<0.001	0.7-0.85
CRP5(mg/L)	0.76	0.05	<0.001	0.67-0.85
CRP5c	0.81	0.03	<0.001	0.75-0.87
PCT1 (ng/mL)	0.57	0.05	0.11	0.49-0.67
PCT3 (ng/mL)	0.61	0.05	0.02	0.52-0.71
PCT3c	0.57	0.04	0.13	0.49-0.66
PCT5 (ng/mL)	0.73	0.04	<0.001	0.65-0.82
PCT5c	0.65	0.05	<0.001	0.57-0.75
CRP1*PCT1	0.55	0.05	0.36	0.45-0.65
CRP3*CRP3c	0.7	0.05	<0.001	0.6-0.8
CRP5*CRP5c	0.77	0.05	<0.001	0.68-0.86
PCT3*PCT3c	0.65	0.04	<0.001	0.56-0.74
PCT5*PCT5c	0.74	0.04	<0.001	0.66-0.83
CRP3*PCT3	0.7	0.05	<0.001	0.6-0.81
CRP3c*PCT3c	0.76	0.04	<0.001	0.68-0.84
CRP5*PCT5	0.79	0.04	<0.001	0.71-0.87
CRP5c*PCT5c	0.67	0.04	<0.001	0.58-0.76
CRP5*CRP5c*PCT5*PCT5c	0.79	0.04	<0.001	0.71-0.87
CRP3*CRP3c* CRP5*CRP5c	0.85	0.04	<0.001	0.77-0.92
PCT3*PCT3c*PCT5*PCT5c	0.81	0.04	<0.001	0.73-0.9
CRP3*CRP3c* CRP5*CRP5c* PCT3*PCT3c*PCT5*PCT5c	0.81	0.04	<0.001	0.73-0.88
CURB-65	0.53	0.05	0.53	0.44-0.63
CRP3*CRP3c*CRP5*CRP5c*CURB-65	0.77	0.04	<0.001	0.69-0.85
PCT3*PCT3c*PCT5*PCT5c*CURB-65	0.72	0.05	<0.001	0.64-0.81

^a^Under the nonparametric assumption

^b^Null hypothesis: true area = 0.5

^c^ROC receiver operating characteristic, AUC area under the curve, SE standard error, CI confidence interval

## Discussions

In accordance with the current literature, the clinical characteristics of the patients included in this study frequently had a comorbidity of respiratory disorders, diabetes mellitus, congestive heart failure and cancer [23]. So far, most studies focused on the diagnostic performance of serum biomarkers, especially CRP and PCT on the pneumonia diagnosis [1, 5, 7, 11, 24, 25]. Only very few research studied the predictive value of serum biomarkers in the pneumonia outcomes [6, 14, 17, 26–28]. A dynamic approach to biomarkers could capture the progression of disease and might be more effective in evaluating pneumonia prognosis.

In this context, we observed serum CRP and PCT levels measured at different time points after admission. The main findings of this study are threefold. First, circulating CRP and PCT levels were significant different in the pneumonia patients infected with different pathogens. However, there was no significance of the serum CRP1 and PCT1 levels between survivors and non-survivors. This indicated that the initial CRP and PCT levels could not provide useful information to assist with mortality prediction in hospitalized CAP patients, which was consistent with the results from previous studies. Previous studies had showed that simply measuring the initial CRP and PCT levels did not improve clinical score for mortality but that following the kinetics of PCT did so [6, 29]. However, Akihiro ITO’s study found that the initial CRP and PCT levels were significant different between survivors and non-survivors [30]. Furthermore, they found that PCT levels on day3/day1 ≥ 1, CRP levels on day1 ≥ 100 mg/L and CURB-65 ≥ 3 were prognostic variables in CAP. The different basic characteristics of research groups in these studies were the main reasons for the different results. The average age in our study was younger than Akihiro ITO’s study (58.53 vs. 73.2), while composition ratio of CURB-65 class was similar (Class 0–2: 82.6 vs. 75.9, Class 3–5: 17.4 vs. 24.1). Similar proportion of CURB-65 in the population aged below and above 65 years old, indicating the more complicate comorbidities or more severe CAP disease in our study which resulting the similar initial CRP and PCT levels between survivors and non-survivors.

Second, consistent with the previous report [6], CRP levels were independent prognostic predictors of CAP clinical outcomes. PCT has been used as a biomarker for initiating or terminating antibiotic therapy in various clinical settings in the previous studies [31, 32]. In this work, we confirmed the predictive role of CRP and PCT in CAP prognosis. Serial serum CRP3, CRP5, PCT3, PCT5 and PCT5c levels were statistically lower in CAP survivors than non-survivors. CRP3c < 0, CRP5c < 0 and PCT5c < 0 were observed with a statistically lower frequency in patients with 30-day mortality and initial treatment failure. Significant predictive ability was found for 30-day mortality with CRP3, CRP5 and PCT5 levels.

Third, there was low level of multicollinearity among each biomarker. The capacity of serial serum biomarkers combined to predict 30-day mortality was higher than only one biomarker or a combination of two of the biomarkers. Though the CRP and PCT clearances were not directly associated with the CAP prognosis, when combined with the serum biomarker levels, the increased AUC-ROC indicated the greater prognosis capacity. This was consistent with previous report [33], CRP kinetics can be used to identify ventilator-associated pneumonia patients with poor outcome. This also highlighted the necessary to measure the values of serum biomarkers serially. However, the combination with CURB65 did not increase the predictive AUC-ROC of serum biomarker.

There were some limitations in our study. Firstly, the missing data for laboratory biomarkers in some patients, potential classification bias in the etiologic diagnosis. However, our evaluation has been done in a large study population even excluding missing data. Secondly, since the average age of the patients in our study was near 60 years old, whether these results are generalizable to CAP patients in children or aged greater than 80 years old needs further evaluation. Finally, the objects studied usually combined with other diseases, which might affect the serum CRP and PCT levels. But the complicated diseases were the true status for most hospitalized CAP patients. Thus, further studies with a prospective design are needed to explore the influence of other comorbidity on the biomarkers level and hospitalized CAP prognosis.

## Conclusions

This is a large and comprehensive study focused on the predictive value of serum dynamic CRP, PCT levels and their clearance in hospitalized CAP outcomes. The low correlations between the two biomarkers and the only moderate prognostic accuracy calls for a head-to-head trial comparing the ability of both markers to monitor the therapeutic effect and to answer the question which marker is superior in the prognosis prediction

### Key messages

The dynamic serum CRP and PCT levels have moderate predictive value on the prognosis of hospitalized CAP.

## Additional files

Additional file 1: **Table S1.** Viral and bacterial data for patients classified as “definite CAP. (DOCX 13 kb)

Additional file 2: **Table S2.** Comparisons of biomarkers characteristics within pneumonia patients infected by different pathogens. (DOCX 15 kb)

## Abbreviations

AUC: Areas under the curve
CAP: Community-acquired pneumonia
CCI: Charlson Comorbidity Index
CI: Confidence interval
CRP: C-reactive protein
CRPc: CRP clearance
PCT: Procalcitonin
PCTc: PCT clearance
ROC: Receiver operating characteristic
VAP: Ventilator- associated pneumonia
WBC: White blood cell count
