# Supplementary material for: The moderate predictive value of serial serum CRP and PCT levels for the prognosis of hospitalized community-acquired pneumonia
Source: Respir Res. 2018 Oct 1;19:193. doi: 10.1186/s12931-018-0877-x (PMC6167901; doi:10.1186/s12931-018-0877-x)
Supplement: Supplementary file 1 — Table S1. Viral and bacterial data for patients classified as “definite CAP. (DOCX 13 kb) [file 12931_2018_877_MOESM1_ESM.docx]

| Supplement Table 1 viral and bacterial data for patients classified as “definite CAP” | |
| --- | --- |
| Viral and bacterial data | Patients classified as definite CAP |
|  | N=350 |
| **Virus identified by multiplex PCR** |  |
| Influenza A virus | 37^a, b^ |
| Influenza B virus | 11 |
| Parainfluenza virus | 13 |
| Rhinovirus | 19^d, e^ |
| Respiratory syncytial virus A | 11 |
| Respiratory syncytial virus B | 11 |
| Metapneumovirus | 12^c^ |
| Adenovirus | 1 |
| **Bacteria identified by multiplex PCR** |  |
| *Mycoplasma pneumoniae* | 12 |
| **Bacteria identified by cytobacteriological examination of sputum, blood culture, urine antigen test** |  |
| *Streptococcus pneumoniae* | 85^a, d^ |
| *Haemophilus influenzae* | 16 |
| *Enterobacteriaceae* | 21 |
| *Legionella pneumonia* | 15 |
| Intracellular bacteria | 22^b^ |
| Miscellaneous | 15 |
| **Negative multiplex PCR and negative bacterial sample** | 39 |
| **Negative multiplex PCR and no bacterial sample performed** | 10 |
| *Note: ^a^ one patient with a co-infection Streptococcus pneumoniae + Influenza A virus, ^b^one patient with a co-infection Intracellular bacteria + Influenza A virus, ^c^one patient with a co-infection* Enterobacteriaceae *+ Influenza A virus, ^d^one patient with a co-infection Streptococcus pneumoniae + Rhinovirus, Miscellaneous: multiple atypical infections.* | |
