# Supplementary material for: The moderate predictive value of serial serum CRP and PCT levels for the prognosis of hospitalized community-acquired pneumonia
Source: Respir Res. 2018 Oct 1;19:193. doi: 10.1186/s12931-018-0877-x (PMC6167901; doi:10.1186/s12931-018-0877-x)
Supplement: Supplementary file 2 — Table S2. Comparisons of biomarkers characteristics within pneumonia patients infected by different pathogens. (DOCX 15 kb) [file 12931_2018_877_MOESM2_ESM.docx]

| Supplement table 2 Comparisons of biomarkers characteristics within pneumonia patients infected by different pathogens | | | | | |
| --- | --- | --- | --- | --- | --- |
| **Variable** | **All subjects** | **Probable bacterial** | **Probable viral** | **Probable fungal** | **Undetermined** |
| Males /Total | (204 /350) | (118/205) | (50 /83) | (19/32) | (14/24) |
| Ages (years) | 58.5±19.1 | 58.3±19.4 | 59.8±18.4 | 57.4±21 | 64±15.5 |
| CRP1 (ng/L) | 65.3±84.7 | 88.9±96.5* | 23.4±24.5* | 24±21.1* | 53.5±78.4* |
| CRP3 (ng/L) | 56.4±77.4 | 71.8±90.9* | 29.7±35.7* | 34.6±29.7* | 43.8±63.6* |
| CRP3c | 122±725 | 81±625* | 238±1079* | 91±206* | 111±416* |
| CRP5 (ng/L) | 44.8±68.5 | 51.4±77.5 | 33.4±51.6 | 29±28.5 | 43.7±63.1 |
| CRP5c | 129±744 | 78±686* | 227±882* | 81±229* | 212±882* |
| PCT1 (ng/mL) | 1.8±7.1 | 2.2±8.6 | 0.8±2.4 | 0.6±2.1 | 2.8±7.1 |
| PCT3 (ng/mL) | 1.7±6.3 | 1.5±5 | 1±2.3 | 0.9±1.6 | 4.2±14.7 |
| PCT3c | 791±2654 | 675±2430 | 798±2527 | 1009±2072 | 1260±4080 |
| PCT5 (ng/mL) | 1.2±3.7 | 1.2±3.2 | 0.7±1.3 | 1±1.6 | 2.4±8.3 |
| PCT5c | 695±2463 | 507±1679 | 650±2080 | 1415±3391 | 1342±4878 |
| WBC1 (10^9^cells/μL) | 10.4±8 | 10.4±7 | 9.7±5 | 7.8±2.5 | 13.2±16.1 |
| WBC3 (10^9^cells/μL) | 9.5±5 | 9.9±5.5 | 8.6±4.2 | 8.8±3.4 | 9.6±4.6 |
| WBC5 (10^9^cells/μL) | 10.5±6.6 | 9.8±6.5 | 11.8±7.4 | 6.6±0.8 | 12.5±6.2 |
| day (days) | 17.7±6.8 | 16.9±7.2* | 17.8±5.7* | 19.8±5.7* | 20.1±6.6* |
| * The mean difference is significant at the 0.05 level. | | | | | |
